# Prevalence of attention-deficit/hyperactivity disorder symptoms and associated factors among university students in Egypt: a cross-sectional study

**DOI:** 10.1038/s41598-026-63835-y

**Published:** 2026-08-25

**Authors:** Sara Hosny El-Farargy, Ahmed Mahmoud Fawzy, Ahmed Samy Elgammal, Hamed Abdelma’aboud Mostafa, Muhammad Ashraf Husain, Hossam Tharwat Ali, Gasm Alseed Abdelmonim, Egyptian Collaborators Group, Dalia Atef, Islam Ali Abdel, Enas Mitwally Awad, Sally Ashraf Emam, Ibrahim Ahmed Ibrahim Fawzy, Alaa Maged Mohammed, Elsaeed Saleh Abdelmonsef, Mohamed Mahmoud Alkarram, Israa Mohamed Khadrah, Doha El-Sayed Ellakwa, Marwa Abdelkader, Ranim Mohamed Farouk Mohamed Hussein Gadalla, Nada Sayed Abdelhamed Ahmed, Doaa Mohammed Alnabwy

**Affiliations:** 1 Faculty of Medicine, Benha University, Banha, Egypt; 2 Faculty of Medicine, Port Said University, Port Said, Egypt; 3 Faculty of Medicine, Tanta University, Tanta, Egypt; 4 Faculty of Medicine, Al-Azhar University, Cairo, Egypt; 5 Qena Faculty of Medicine, South Valley University, Qena, Egypt; 6 Shendi University Faculty of Medicine, Shendi, Sudan; 7 Faculty of Medicine, Alexandria University, Alexandria, Egypt; 8 Benha Faculty of Medicine, Banha, Egypt; 9 Faculty of Medicine, Suez University, Suez, Egypt; 10 Faculty of Physical Therapy, Benha University, Banha, Egypt; 11 Faculty of Medicine, South Valley University, Qena, Egypt; 12 Faculty of Medicine, Mansoura University, Mansoura, Egypt; 13 Damietta Faculty of Medicine, Al-Azhar University, Cairo, Egypt; 14 Alexandria Faculty of Medicine, Alexandria, Egypt; 15 Department of Biochemistry and Molecular Biology, Faculty of Pharmacy for Girls, Al-Azhar University, Cairo, Egypt; 16 Department of Biochemistry, Faculty of Pharmacy, Sinai University, Kantra Branch, Ismailia, Egypt; 17 Faculty of Medicine, Mansoura University, Manchester Medical Program, Mansoura, Egypt; 18 October 6 University Faculty of Medicine, 6th of October City, Egypt; 19 Faculty of Medicine for Girls, Al Azhar University, Cairo, Egypt

**Keywords:** ADHD, Prevalence, University students, Egypt, Associated factors, Mental health, ASRS, Diseases, Health care, Medical research, Neuroscience, Psychology, Psychology, Risk factors

## Abstract

Attention-Deficit/Hyperactivity Disorder (ADHD) is a prevalent neurodevelopmental disorder that persists in adulthood, affecting academic performance and daily functioning. Despite its significance, limited research has explored the prevalence of ADHD-like symptoms among university students in Egypt. This study aims to estimate the prevalence of ADHD Symptoms among Egyptian university students and identify associated factors. A cross-sectional study was conducted using an online self-administered questionnaire among students aged ≥ 18 years or older from various Egyptian universities. The Adult ADHD Self-Report Scale (ASRS) was used to assess ADHD symptoms. A non-probability convenience sampling technique was applied, and multivariable logistic regression was performed to determine associated risks of ADHD positivity. A total of 3,136 responses were collected, with 2,383 valid responses included in the final analysis. The estimated prevalence of ADHD SYMPTOMS was 37.9% (95% CI: 36.0%–39.9%), obesity (OR = 2.09, 95% CI: 1.14–3.95, *p* = 0.019), smoking (OR = 1.93, 95% CI: 1.25–3.01, *p* = 0.003), sleep disorders (OR = 2.12, 95% CI: 1.77–2.55, *p* < 0.001), childhood trauma (OR = 1.42, 95% CI: 1.18–1.71, *p* < 0.001), and history of mental health conditions (OR = 1.71, 95% CI: 1.34–2.18, *p* < 0.001). Regular exercise was associated with lower odds of ADHD (OR = 0.55, 95% CI: 0.44–0.70, *p* < 0.001). Prevalence of ADHD-like symptoms among Egyptian university students is notably high, with multiple lifestyle and psychosocial factors showing significant associations with its occurrence. These findings highlight the need for increased awareness, early screening, and targeted interventions to support affected students. Future research should further investigate the cultural and educational influences on ADHD in Egypt.

## Introduction

Attention Deficit Hyperactivity Disorder (ADHD) is a pervasive neurodevelopmental challenge with global implications, impacting approximately 5–10% of school-aged children and persisting into adulthood in up to 60% of cases^1^. The enduring nature of ADHD poses significant challenges, affecting not only immediate symptoms but also academic performance, interpersonal relationships, and overall quality of life^2^. Adults grappling with ADHD encounter hurdles in crucial aspects of daily functioning, including organization, time management, and impulse control, influencing both professional and personal spheres^2^. ADHD has garnered global attention for its prevalence and repercussions on educational outcomes. Studies conducted across diverse cultural contexts underline its association with academic underachievement and psychosocial difficulties^3^. However, these findings primarily stem from Western-centric research, leaving a void when considering the unique cultural and educational dynamics within Egyptian universities.

According to recent data from Egyptian medical students, there is a noteworthy negative link between academic achievement (GPA) and Adult ADHD Self-Report Scale (ASRS) scores, and the incidence of ADHD symptoms may exceed 11%. This validates the substantial symptom burden seen in our sample, especially among students in demanding educational settings^4^.

Although ADHD was once thought to be a childhood condition, longitudinal statistics show that between 40 and 50% of cases continue into adulthood. Overt hyperactivity frequently gives way to interior restlessness and severe executive dysfunction during this transition, which poses particular difficulties for college students^5^.

Egyptian higher education institutions serve as a distinct backdrop, shaped by cultural nuances and academic structures unique to the region. The need to explore ADHD within this specific context becomes imperative, given potential variations in symptom manifestation and the influence of cultural expectations on academic performance^6^. As research has extensively explored ADHD in children and adolescents, a growing interest emerges in understanding its prevalence and associated factors among university students^7^. While global studies underscore the persistence of ADHD symptoms into adulthood, the unique context of Egypt, with its distinct cultural and environmental factors, necessitates a comprehensive investigation^8^. Limited research on ADHD prevalence among university students in Egypt prompts a focused exploration to bridge this knowledge gap. Moreover, the need to identify associated factors becomes crucial for a nuanced understanding of ADHD in university settings. Factors such as genetic predisposition, environmental influences, and lifestyle considerations may contribute to ADHD symptomatology in this unique population^2, 8, 9^.

There is a dearth of multi-institutional data examining ADHD in both medical and non-medical fields, despite the fact that earlier Egyptian research on the disorder has mostly concentrated on child populations or small single-center university samples. By offering a more comprehensive evaluation and distinctively investigating recently incorporated behavioral and lifestyle factors unique to this demographic, our study fills this gap.

This research endeavors to address the gap in our understanding by investigating the prevalence of ADHD and identifying associated factors among students in Egyptian universities. The significance of this study extends beyond academia, offering insights that can inform educational practices and policies in the Egyptian higher education and public health systems.

## Methods

### Study design

This is a descriptive, cross-sectional study that included students at Egyptian universities aged 18 years or older who gave consent to participate. The study was conducted between November 2023 and February 2024, using an online questionnaire.

### Sampling strategy

A non-probability convenience sampling technique was used. The minimum required sample size was calculated based on the single proportion formula using data from previous Egyptian literature. Assuming an expected the prevalence of ADHD Symptoms rate of 2.66% among Egyptian university students (based on a previous study at Tanta University by Abd El-Hay et al.), an absolute margin of error (deviation) of 1% (0.01), a 95% confidence level, and a total higher education student population size of 3,495,100 (according to the Central Agency for Public Mobilization and Statistics [CAPMAS])^37,38^, the minimum required sample size was determined to be 2,388 participants.

### Study tool

The online questionnaire consisted of two sections with a total of 37 questions. The first section included the sociodemographic characteristics: age, height, weight, gender, university, faculty, marital status, nationality, socioeconomic status, family history of ADHD, geographic location (from urban OR rural area), history of ADHD, time spent on social media in hours, time spent on electronic devices in hours, history of pediatric trauma, history of any chronic disease or drugs, history of any psychiatric disorder, the use of any addictive substances.

The second section was the Adult ADHD Self-Report Scale (ASRS), a previously validated scale that is consistent with the ADHD criteria of the Diagnostic and Statistical Manual of Mental Disorders (DSM), with an established sensitivity of 68.7%–100% and specificity of 83.3%–99.5%^10–14^. A rigorous forward-backward translation protocol was independently conducted by two professional bilingual translators, followed by expert panel harmonization for cultural adaptation. The ASRS scale consisted of three subscales: the inattentive subscale, the hyperactive/impulsive subscale (motor), and the hyperactive/impulsive subscale (verbal), with a total of 18 questions. Participants were asked to answer each question with (never, rarely, sometimes, often, or very often).

Participants with scores of at least four in the first six items (part A) were considered to have symptoms that are highly consistent with an ADHD diagnosis in adults, while scores of the rest of the questions were to provide additional cues for the severity of the participants’ symptoms and their impact on their lives.

### Data collection

Data collection was conducted through the online survey tool “Google Forms”. Participants were approached by data collectors who invited them to fill out the questionnaire and explained some statements when needed. This study’s target population included undergraduate students currently enrolled in Egyptian universities. During the online data collection phase, a number of quality control measures were put in place to guarantee data integrity and avoid automated or duplicate responses. Google Forms was used to enable the “Limit to 1 response” function, which requires a working Google login in order to prevent repeated entries from the same participant. To further filter out automated bots, a CAPTCHA verification step was incorporated. A very dependable final dataset was ensured by subsequent data cleaning, which verified the lack of incorrect entries, matching timestamps, or straight-lining response patterns”.

Inclusion criteria were: (1) full-time students, (2) aged 18 years or older, and (3) willing to provide informed consent. Exclusion criteria were: (1) Students with severe, uncontrolled neurological or mental conditions (such as schizophrenia, active psychosis, epilepsy, or significant cognitive impairments) that would clinically affect their ability to understand or independently complete the online questionnaire were excluded, and (2) incomplete or inconsistent survey responses, which were removed to ensure data integrity.” With confidentiality being assured. A pilot study (n = 69) was conducted to assess the clarity of questions in the Arabic version. In the current study, the Arabic version of the ASRS showed high internal consistency, with a Cronbach’s alpha coefficient of 0.85. The final analysis did not include the responses of the pilot study.

### Ethical considerations

Ethical approval was obtained from the Ethics Committee of Benha University before starting the collection of data. Additionally, a checkbox consent was obtained from each participant before filling out the questionnaire, and after a comprehensive explanation of the aim of the study, emphasizing the complete confidentiality of the participants’ data.

### Statistical analysis

Data analysis was conducted using R version 4.3.1 (2023-06-16 ucrt). Descriptive statistics were computed to summarize participant characteristics. Categorical variables (e.g., sex, socioeconomic status) were reported as frequencies and percentages, while continuous variables (e.g., age, BMI, sleep duration) were summarized as means ± standard deviations (SD) or medians with interquartile ranges (IQR) for non-normally distributed variables. The prevalence of ADHD symptoms was calculated as a proportion with a 95% confidence interval (CI) using the Wilson score method.

Univariable logistic regression models were used to assess associations between demographic, lifestyle, and clinical variables and ASRS positivity. Variables included sex, college type, BMI categories (underweight, normal, overweight, obese), smoking status, exercise frequency, sleep disorders, social media use duration, and history of childhood trauma or chronic illness. Adjusted odds ratios (OR) with 95% CIs and p-values were reported. To find independent correlates of ASRS positive using a backward stepwise selection procedure, clinically significant variables and variables that showed statistical significance (*p* < 0.05) in the univariable analysis were added to a multivariable logistic regression model. The Variance Inflation Factor (VIF) was used to evaluate multicollinearity among the independent variables before modeling, making sure that all values were less than 2.5. The Hosmer-Lemeshow test was used to assess the final model’s goodness-of-fit; a p-value > 0.05 indicated an adequate model fit. The results were presented as adjusted Odds Ratios (aOR) with 95% Confidence Intervals (CI) after the final model was adjusted for variables. Categorical variables were dummy-coded with reference groups specified as follows: underweight (BMI < 18.5) for BMI comparisons, female for sex, and non-smokers for smoking status. The threshold for statistical significance was established at 0.05 (two-tailed).

## Results

**Fig. 1:**
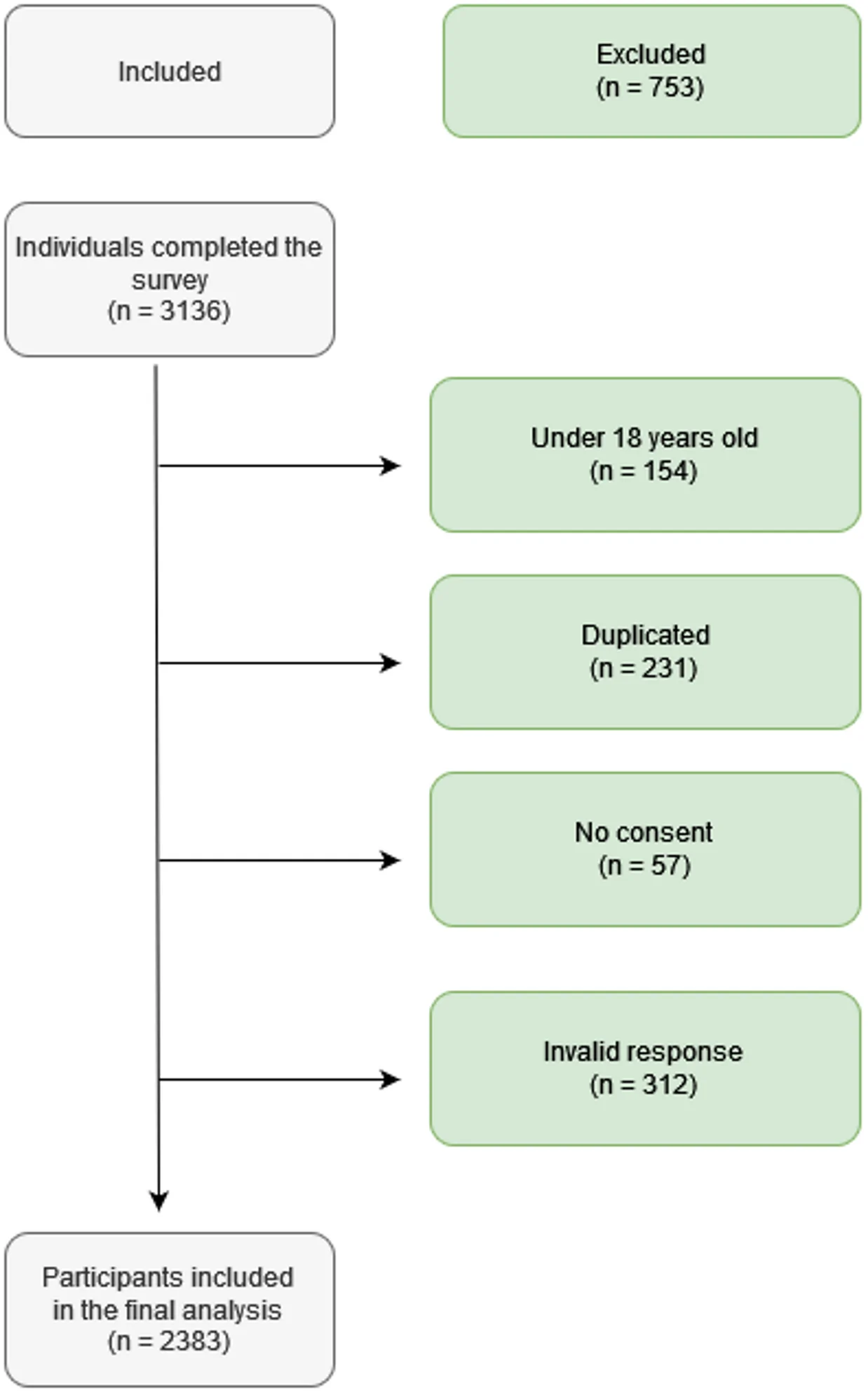
Flowchart showing the inclusion and exclusion process of participants. A comparative analysis of behavioral habits, lifestyle factors, and substance use between the screening-negative group (ASRS) and screening-positive ADHD group (ASRS).

A total of 3136 responses were collected for the study. After applying the exclusion criteria — including participants under 18 years old, duplicated submissions, responses without consent, and invalid or incomplete entries—753 responses were removed from the dataset. The final dataset consisted of 2383 valid responses, which were included in the statistical analysis, yielding a questionnaire completion/response rate of 76.0% (Fig. 1).

### Sample characteristics

The majority of participants were young adults aged 18–24 years (97%, *n* = 2313), with a mean age of 21 years (SD = 2). Females constituted 65% of the sample (*n* = 1538). Nearly all participants were Egyptian nationals (97%, *n* = 2321) and unmarried (97%, *n* = 2300). Regarding socioeconomic status, 60% (*n* = 1423) reported having just enough income, while 5.6% (*n* = 134) reported insufficient income. Urban residents accounted for 53% (*n* = 1274) of participants. Regionally, the Delta region was the most represented (36%, *n* = 868), followed by Upper Egypt (10%, *n* = 250) and the Suez Canal region (6.5%, *n* = 154). Most participants were enrolled in public universities (85%, *n* = 2026) and medical-related colleges (74%, *n* = 1754). The mean body mass index (BMI) was 25.0 (SD = 4.4), with 54% (*n* = 1289) in the normal BMI range and 12% (*n* = 275) classified as obese (Table 1).

**Table 1:** Demographic characteristics among the study participants.

Variable	Total, *n* (%)^1^	ADHD screening		Univariable analysis		
		Negative, *n* (%)^2^	Positive, *n* (%)^3^	OR^4,5^	95% CI^5^	*p*-value
Age						
Youth (15–24 years)	2313 (97)	1439 (97)	874 (97)	–	–	
Young adults (25–44 years)	70 (2.9)	40 (2.7)	30 (3.3)	1.23	0.76-1.99	0.390
Mean (±SD)	21 (±2)	21 (±2)	21 (±2)			
Median [Q1-Q3]	21 [19-22]	21 [19-22]	20 [19-22]			
Sex						
Female	1538 (65)	919 (62)	619 (68)	–	–	
Male	845 (35)	560 (38)	285 (32)	0.76**	0.63-0.90	0.002
Nationality						
Egyptian	2321 (97)	1444 (98)	877 (97)	–	–	
Non-Egyptian	62 (2.6)	35 (2.4)	27 (3.0)	1.27	0.76-2.11	0.357
Marital status						
Currently married	83 (3.5)	52 (3.5)	31 (3.4)	–	–	
Currently not married	2300 (97)	1427 (96)	873 (97)	1.03	0.66-1.63	0.911
Family income						
Just enough	1423 (60)	877 (59)	546 (60)	–	–	
More than enough	826 (35)	524 (35)	302 (33)	0.93	0.77-1.10	0.394
Not enough	134 (5.6)	78 (5.3)	56 (6.2)	1.15	0.80-1.65	0.437
Residence place						
Rural	1109 (47)	695 (47)	414 (46)	–	–	
Urban	1274 (53)	784 (53)	490 (54)	1.05	0.89-1.24	0.570
Region						
Greater Cairo	524 (22)	325 (22)	199 (22)	–	–	
Alexandria	587 (25)	389 (26)	198 (22)	0.83	0.65-1.06	0.141
Delta	868 (36)	518 (35)	350 (39)	1.10	0.88-1.38	0.386
Suez Canal	154 (6.5)	92 (6.2)	62 (6.9)	1.10	0.76-1.59	0.609
Upper Egypt	250 (10)	155 (10)	95 (11)	1.00	0.73-1.36	0.995
University						
Public	2026 (85)	1271 (86)	755 (84)	–	–	
Private	357 (15)	208 (14)	149 (16)	1.21	0.96-1.51	0.109
College						
Medical-related college	1754 (74)	1066 (72)	688 (76)	–	–	
Not medical-related college	629 (26)	413 (28)	216 (24)	0.81*	0.67-0.98	0.030
BMI						
Underweight (BMI < 18.5)	56 (2.3)	39 (2.6)	17 (1.9)	17 (1.9)	–	
Normal (18.5 ≤ BMI < 25)	1289 (54)	817 (55)	472 (52)	472 (52)	0.75-2.43	0.342
Overweight (25 ≤ BMI < 30)	763 (32)	479 (32)	284 (31)	284 (31)	0.77-2.51	0.305
Obese (BMI ≥ 30)	275 (12)	144 (9.7)	131 (14)	131 (14)	1.14-3.95	0.019
Mean (±SD)	25.0 (±4.4)	24.7 (±4.2)	25.4 (±4.7)	25.4 (±4.7)		
Median [Q1-Q3]	24.4 [22.0-27.1]	24.2 [21.8-26.8]	24.5 [22.2-27.7]	24.5 [22.2-27.7]		

^1^Total count (*N* = 2383).

^2^Negative count (*N* = 1479).

^3^Positive count (*N* = 904).

^4^*p<0.05; **p<0.01; ***p<0.001.

^5^OR = odds ratio, CI = confidence interval.

### Lifestyle and history characteristics

A majority of participants were non-smokers (96%, *n* = 2299) and did not engage in regular exercise (79%, *n* = 1879). Sleep disorders were reported by 44% (*n* = 1052). The average daily sleep duration was 7.28 h (SD = 1.66), with 47% (*n* = 1125) sleeping 6–8 h and 33% (*n* = 785) sleeping less than 6 h. Daily social media use was highest among those reporting 4 + hours (47%, *n* = 1112), with a median of 4 h (IQR: 3–6). Time spent on electronic devices for purposes other than social media was most commonly reported as 0–2 h (42%, *n* = 1003), with a median of 4 h (IQR: 2–7). Childhood trauma or impactful events were reported by 58% (*n* = 1381). Chronic illnesses were uncommon (11%, *n* = 252). A past diagnosis of mental health conditions was reported by 15% (*n* = 357), and 3.9% (*n* = 93) reported a prior diagnosis of ADHD by a doctor. Regular medication use was noted in 19% (*n* = 448), while drug or stimulant use was rare (Table 2).

**Table 2:** Lifestyle and history among the study participants.

Variable	Total, *n* (%)	ADHD screening		Univariable analysis		
		Negative, *n* (%)	Positive, *n* (%)	OR^1,2^	95% CI^2^	*p*-value
Smoking						
No	2299 (96)	1440 (97)	859 (95)	–	–	
Yes	84 (3.5)	39 (2.6)	45 (5.0)	1.93**	1.25-3.01	0.003
Regular exercise						
No	1879 (79)	1111 (75)	768 (85)	–	–	
Yes	504 (21)	368 (25)	136 (15)	0.53***	0.43-0.66	<0.001
Sleep disorders						
No	1331 (56)	959 (65)	372 (41)	–	–	
Yes	1052 (44)	520 (35)	532 (59)	2.64***	2.23-3.13	<0.001
Average daily sleep hours last month						
6–8 h	1125 (47)	735 (50)	390 (43)	–	–	
< 6 h	785 (33)	462 (31)	323 (36)	1.32**	1.09-1.59	0.004
9–10 h	404 (17)	247 (17)	157 (17)	1.20	0.95-1.51	0.132
> 10 h	69 (2.9)	35 (2.4)	34 (3.8)	1.83*	1.12-2.99	0.015
Mean (±SD)	7.28 (±1.66)	7.30 (±1.59)	7.25 (±1.78)			
Median [Q1-Q3]	7.00 [6.00-8.00]	7.00 [6.00-8.00]	7.00 [6.00-8.00]			
Daily hours spent on social media						
Moderate Usage (2–3 h)	868 (36)	552 (37)	316 (35)	–	–	
Low Usage (0–1 h)	403 (17)	301 (20)	102 (11)	0.59***	0.45-0.77	<0.001
High Usage (4+ hours)	1112 (47)	626 (42)	486 (54)	1.36**	1.13-1.63	0.001
Mean (±SD)	5 (±3)	5 (±3)	5 (±3)			
Median [Q1-Q3]	4 [3-6]	4 [3-6]	5 [3-7]			
Daily hours spent on electronic devices for other purposes						
3–4 h	711 (30)	454 (31)	257 (28)	–	–	
0–2 h	1003 (42)	648 (44)	355 (39)	0.97	0.79-1.18	0.749
4+ hours	669 (28)	377 (25)	292 (32)	1.37**	1.10-1.70	0.004
Mean (±SD)	5.0 (±3.5)	4.8 (±3.5)	5.3 (±3.6)			
Median [Q1-Q3]	4.0 [2.0-7.0]	4.0 [2.0-7.0]	5.0 [2.0-8.0]			
Childhood trauma or impactful events						
No	1002 (42)	706 (48)	296 (33)	–	–	
Yes	1381 (58)	773 (52)	608 (67)	1.88***	1.58-2.23	<0.001
Chronic illness status						
No	No	2131 (89)	1358 (92)	773 (86)	–	–
Yes	Yes	252 (11)	121 (8.2)	131 (14)	1.46-2.47	1.46-2.47
History of diagnosed mental health conditions						
No	No	2026 (85)	1318 (89)	708 (78)	–	–
Yes	Yes	357 (15)	161 (11)	196 (22)	1.81-2.85	1.81-2.85
Family history of ADHD						
No	No	2164 (91)	1363 (92)	801 (89)	–	–
Yes	Yes	219 (9.2)	116 (7.8)	103 (11)	1.14-2.00	1.14-2.00
Regular medication use for chronic diseases						
No	No	1935 (81)	1243 (84)	692 (77)	–	–
Yes	Yes	448 (19)	236 (16)	212 (23)	1.31-1.99	1.31-1.99
Use of addictive drugs or illicit stimulants						
No	No	2347 (98)	1464 (99)	883 (98)	–	–
Yes	Yes	36 (1.5)	15 (1.0)	21 (2.3)	1.20-4.61	1.20-4.61

^1^*p<0.05; **p<0.01; ***p<0.001.

^2^OR = Odds Ratio, CI = Confidence Interval.

*Regular exercise is defined as ≥150 min of moderate physical activity/week according to WHO criteria.

*Family history is restricted to first-degree relatives only.

*Childhood trauma is evaluated as a binary variable (exposure vs. non-exposure).

### ASRS responses and prevalence of ADHD-like symptoms

ASRS responses varied across items. In part A, common difficulties included trouble completing projects (37.2% “sometimes”) and organizing tasks (35.6% “sometimes”). In part B, frequent issues included difficulty focusing on repetitive tasks (32.4% “sometimes”) and being easily distracted by noise or activity (30.5% “sometimes”) (Table 3).

**Table 3:** Responses of participants on the adult ADHD Self-Report Scale (ASRS).

Questions	Never	Rarely	Sometimes	Often	Very Often
Part A					
1. Trouble finishing project details	394 (16.5%)	441 (18.5%)	887 (37.2%)	400 (16.8%)	261 (11%)
2. Difficulty organizing tasks	296 (12.4%)	564 (23.7%)	848 (35.6%)	420 (17.6%)	255 (10.7%)
3. Forgetting appointments	329 (13.8%)	745 (31.3%)	727 (30.5%)	329 (13.8%)	253 (10.6%)
4. Avoiding complex tasks	246 (10.3%)	489 (20.5%)	793 (33.3%)	496 (20.8%)	359 (15.1%)
5. Fidgeting during prolonged sitting	225 (9.4%)	407 (17.1%)	653 (27.4%)	475 (19.9%)	623 (26.1%)
6. Feeling overly active or driven	461 (19.3%)	627 (26.3%)	753 (31.6%)	329 (13.8%)	213 (8.9%)
Part B					
7. Careless mistakes on boring tasks	432 (18.1%)	663 (27.8%)	746 (31.3%)	315 (13.2%)	227 (9.5%)
8. Difficulty focusing on repetitive work	169 (7.1%)	384 (16.1%)	771 (32.4%)	609 (25.6%)	450 (18.9%)
9. Trouble concentrating during conversations	417 (17.5%)	740 (31.1%)	696 (29.2%)	318 (13.3%)	212 (8.9%)
10. Losing or misplacing items	311 (13.1%)	716 (30%)	743 (31.2%)	345 (14.5%)	268 (11.2%)
11. Easily distracted by noise/activity	141 (5.9%)	351 (14.7%)	728 (30.5%)	640 (26.9%)	523 (21.9%)
12. Leaving a seat in expected situations	686 (28.8%)	778 (32.6%)	578 (24.3%)	212 (8.9%)	129 (5.4%)
13. Feeling restless or fidgety	113 (4.7%)	396 (16.6%)	818 (34.3%)	553 (23.2%)	503 (21.1%)
14. Difficulty relaxing in free time	471 (19.8%)	709 (29.8%)	654 (27.4%)	321 (13.5%)	228 (9.6%)
15. Talking excessively in social settings	289 (12.1%)	597 (25.1%)	777 (32.6%)	410 (17.2%)	310 (13%)
16. Interrupting others in conversation	408 (17.1%)	745 (31.3%)	732 (30.7%)	308 (12.9%)	190 (8%)
17. Trouble waiting for your turn	311 (13.1%)	584 (24.5%)	723 (30.3%)	445 (18.7%)	320 (13.4%)
18. Interrupting others while busy	497 (20.9%)	896 (37.6%)	582 (24.4%)	247 (10.4%)	161 (6.8%)

**Fig. 2:**
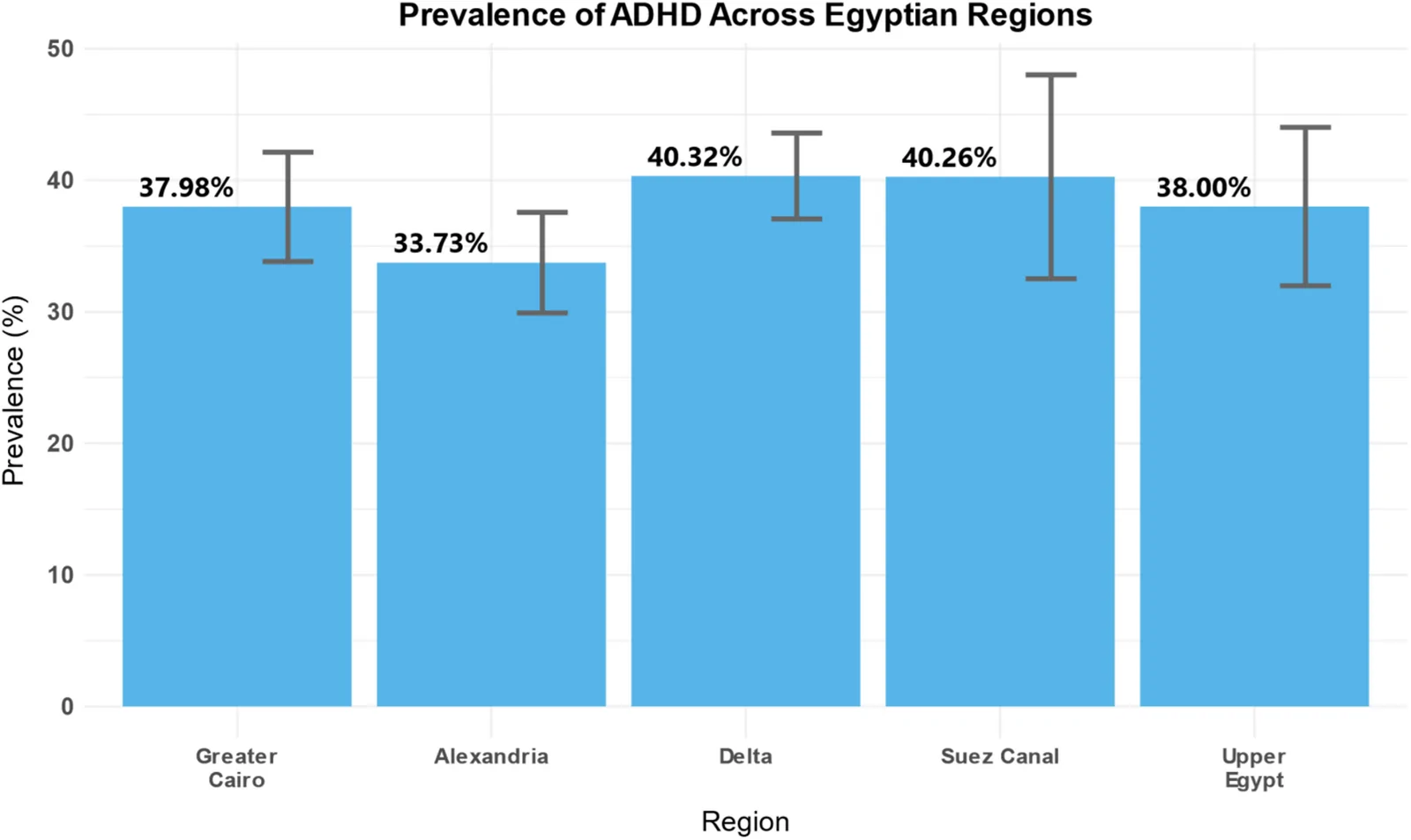
Prevalence of ADHD in different regions of Egypt (Each bar represents the prevalence, and the error 5 bar represents the 95% confidence interval.

To classify participants as positive for ADHD Symptoms, the criteria focus on responses in Part A of the ASRS. Specifically, if four or more marks appear in the darkly shaded boxes of Part A, the individual is considered positive for symptoms highly consistent with ADHD in adults, warranting further investigation. While Part B responses provide additional insight into symptom patterns, the six questions in Part A are the most predictive and serve as the primary screening tool. The prevalence of ADHD Symptoms was 37.9% (95% CI: 36.0% − 39.9%). Figure 2 displays the prevalence of ADHD Symptoms in different regions.

While the ASRS scale identified a current symptom screening prevalence of [37% or your exact number], it is critical to note that this reflects positive screening for elevated symptoms rather than a formal clinical diagnosis. However, among the distinct subgroups of participants who entered the study with a self-reported, prior lifetime clinical diagnosis of ADHD, the majority indicated that their initial professional diagnosis was established during childhood or early adolescence, consistent with the typical developmental onset of the disorder.

#### Univariable analysis

The details of the univariable analysis of demographics and ASRS results are shown in Table 1. Males were less likely to be ASRS-positive compared to females (OR = 0.76, 95% CI: 0.63–0.90, *p* = 0.002). Participants in nonmedical colleges had lower odds of positivity (OR = 0.81, 95% CI: 0.67– 0.98, *p* = 0.03). Obese participants (BMI ≥ 30) had higher odds of positivity compared to underweight participants (OR = 2.09, 95% CI: 1.14 − 3.95, *p* = 0.019).

Regarding lifestyle details (Table 2), smokers had nearly double the odds of positivity (OR = 1.93, 95% CI: 1.25– 3.01, *p* = 0.003). Regular exercisers had lower odds of positivity (OR = 0.53, 95% CI: 0.43–0.66, *p* < 0.001). Participants with sleep disorders had significantly higher odds (OR = 2.64, 95% CI: 2.23–3.13, *p* < 0.001). High usage (4 + hours) of social media increased odds of positivity (OR = 1.36, 95% CI: 1.13–1.63, *p* = 0.001).

### Multivariable logistic regression

Table 4 shows the significant predictors of ASRS positivity. History of mental health conditions (OR = 1.71, CI: 1.34–2.18, *p* < 0.001), sleep disorders (OR = 2.12, 95% CI: 1.77–2.55, *p* < 0.001), childhood trauma (OR = 1.42, 95% CI: 1.18–1.71, *p* < 0.001), and chronic illness (OR = 1.53, 95% CI: 1.15–2.02, *p* = 0.003) correlate with having ADHD according to our self-administered scale while regular exercise reduced such factor (OR = 0.55, 95% CI: 0.44–0.70, *p* < 0.001). Factors such as sex, family history of ADHD, and extreme sleep duration (> 10 h) were not significant in the multivariable model; female sex and family history showed significant associations in univariable analysis .

**Table 4:** Multivariate regression analysis of positive results on ASRS.

Variable	OR^1,2^	95% CI^2^	*p*-value
(Intercept)	0.32***	0.25-0.40	<0.001
Sex			
Female	–	–	
Male	0.99	0.82-1.20	0.949
Family history of ADHD			
No	–	–	
Yes	1.25	0.93-1.69	0.136
History of diagnosed mental health conditions			
No	–	–	
Yes	1.71***	1.34-2.18	<0.001
Regular exercise			
No	–	—	
Yes	0.55***	0.44-0.70	<0.001
Sleep disorders			
No	–	–	
Yes	2.12***	1.77-2.55	<0.001
Average daily sleep hours last month			
6–8 h	–	–	
< 6 h	1.04	0.85-1.28	0.681
9–10 h	0.96	0.74-1.23	0.729
> 10 h	1.30	0.77-2.18	0.330
Daily hours spent on social media			
Moderate usage (2–3 h)	–	–	
Low usage (0–1 h)	0.62***	0.47-0.81	<0.001
High usage (4+ hours)	1.26*	1.04-1.53	0.017
Childhood trauma or impactful events			
No	–	–	
Yes	1.42***	1.18-1.71	<0.001
Chronic illness status			
No	–	–	
Yes	1.53**	1.15-2.02	0.003

^1^*p<0.05; **p<0.01; ***p<0.001; ^2^OR = Odds Ratio, CI = Confidence Interval.

## Discussion

ADHD is a neurodevelopmental disorder characterized by inattention, hyperactivity, and impulsiveness. It is one of the most common childhood disorders, affecting about 1 in 9 US children (11.4%)^15^. While ADHD is often thought to be a childhood disorder, it can persist into adulthood in up to 90% of cases of ADHD^16^. In adults, comorbidities are the rule, with at least one reported in 80% of cases^17–19^. These findings underscore that adult ADHD may cause functional impairment, increasing social burden^20^. To the best of our knowledge, this is the first paper to investigate adult ADHD prevalence among students in Egypt.

Our results showed that ADHD Symptom prevalence was 37.9% in our sample of 2383 participants, with insignificant differences among geographical regions. The high screening positivity rate (37.9%) likely reflects the demographic skew of our sample, where 74% are medical and health sciences students. Compared to most international clinical estimates for adult ADHD, the current study’s screening positivity rate for ADHD symptoms (37.9%) is significantly higher. This high percentage, however, should be regarded cautiously because it indicates screening positivity rather than an actual clinical prevalence in the population. Numerous methodological and contextual factors can be blamed for this exaggeration. First, convenience sampling was used in our online recruitment, which creates a bias toward self-selection. It’s possible that students with mild cognitive, organizational, or attentional difficulties were more inclined to take part in a survey that specifically addressed these symptoms. Second, the ASRS-v1.1 is a highly sensitive screening tool designed to capture current distress, but it lacks the clinical specificity of a formal, structured diagnostic interview. Importantly, the overrepresentation of medical and health-related students—who made up 74% of our sample—is a significant contributor to this high screening positivity. Medical students are frequently subjected to extreme academic pressure, persistent sleep deprivation, burnout, and cumulative weariness, as has been well described in the literature. Clinical overlap exists between the self-reported items on the ASRS-v1.1 scale and the psychological and cognitive symptoms of severe academic stress, such as trouble managing everyday tasks, restlessness, and decreased concentration. Therefore, rather than indicating a clear case of neurodevelopmental ADHD, this substantial symptom overlap with disorders including anxiety, melancholy, and burnout probably inflated the positive screening scores, indicating a high burden of attentional distress among this stressed student group.

We must mention that there were significant associations between a history of mental health problems, childhood trauma, chronic illness, irregular exercise, and sleep disorders, and the likelihood of screening positive for ADHD. We did not find any statistically significant association between sex, family history of ADHD, and extreme sleep duration (> 10 h) in the multivariable model and the prevalence of suspected ADHD cases, however, this association lost significance in multivariable analysis, lifestyle and psychosocial factors included in the final model may have confused the observed link in univariable analysis, as suggested by the loss of significance following multivariable correction.

Sleep disturbances were found to be the main factor of a positive ADHD symptoms screening in our study (OR = 2.12, *p* < 0.001). This result is probably caused by the reciprocal relationship between sleep and attention; long-term sleep deprivation, which is common in our sample of medical students, can impair executive functioning and mirror symptoms of ADHD. On the other hand, impulsivity associated with ADHD frequently results in delayed sleep onset, which exacerbates the symptoms under intense academic pressure.

In our study, we found that the prevalence of ADHD-like symptoms was 37.9%. According to a recent study, 11% of Egyptian medical students tested positive for ADHD symptoms, and there was a strong correlation between the severity of the symptoms and lower academic achievement^4^. According to studies conducted among university students in Saudi Arabia and Jordan, screening positivity rates using the ASRS tool were 40.3% and 24.8%, respectively^21,22^. This reflects a larger regional trend where high symptom prevalence is frequently observed in academic settings. These results highlight how crucial it is to look into ADHD symptoms in a variety of university demographics in order to determine the scope of this problem in the Middle East. When we talk about percentage In Malaysia, Woon and Zakaria reported a prevalence of 15.8%^23^. On the other hand, a Norwegian study reported a prevalence of 2.4% among adults^24^, while another study in Norway also showed a prevalence of 1.1% in a large Norwegian sample (61,129 out of 5,490,678)^25^. In the United states Paper found a prevalence of 20% (68 out of 335)^26^. In a South Korean epidemiological study, results showed a prevalence of 1.1%^27^. In another study Some authors reported a prevalence of 2.9% for people who completed the full criteria and 16.4% for those who partially completed it^23^. Also, in a sample of 966 participants with a mean age of 35.9, and a male/female ratio of 48/52, they noticed an association between Adults’ ADHD and lower educational level and employment status^7^. While another study investigated the prevalence in university students through three different samples (Italy:197, US:799, New Zealand: 213 participants). The prevalence varied from 0% (Italian females) to 8.1% (New Zealand males)^28^.

In the Netherlands study noticed that the adult Prevalence of ADHD symptoms was 1.0% (95% CI 0.6 − 1.6) and 2.5% (1.9–3.4) using a cutoff of six and four current symptoms, respectively^29^. Some authors investigated prevalence in more selective samples, such as psychiatric nonpsychotic adult outpatients and non-clinical participants, reporting it as 16.80% and 5.37%, respectively^30^. In a community-based study, authors found that the prevalence of adult ADHD Symptoms was 4.7% in 720 participants^31^. In the University of Wisconsin-Madison, Heiligenstein et al. reported an adult Prevalence of ADHD symptoms of 4% in 448 college students^32^. Also, Kessler et al. 2006 showed that the prevalence was 4.4% in a sample of 154 participants^13^. In another multinational cross-sectional study in ten countries found that prevalence ranged from 1.2% to 7.3% with an average of 3.4%, with higher prevalence in high-income countries (4.2%)^20^.

This heterogeneity in estimating the prevalence can be attributed to differences in the study methodology and diagnostic criteria of adult ADHD. Variations in methodology include different sample sizes, different populations, non-representative samples, selective population as an outpatient clinic or community-based populations, different diagnostic criteria, different inclusion and exclusion criteria, and different tools to assess ADHD symptoms such as ASRS, Conner’s Adult ADHD Diagnostic Interview for DSM-IV (CAADID), and Wender Utah Rating Scale (WURS).

While our results did not demonstrate an association of suspected ADHD, a negative association between age & ADHD reported in several studies suggests that the clinical presentation of adult ADHD may be different from children. Thus, these findings may indicate the establishment of a unique diagnostic criterion for adult ADHD^7,13,20,31,33,34^.

Our study showed a significantly higher proportion of female participants was observed in the suspected ADHD group compared to the control group (68% vs. 32%). This result is inconsistent with other studies suggesting the male-to-female ratio was 3:1, and some authors reported it as a 10:1 ratio. The discrepancy in the prevalence of ADHD-like symptoms among males compared to established research findings could be attributed to several factors.

There are two primary causes of this gender disparity. First, it is consistent with current enrollment patterns in Egyptian medical and health-related universities, where female students consistently make up a sizable majority of the student body. Second, compared to their male counterparts, female students typically show far higher response rates and a stronger desire to participate in self-reported health and psychological studies, according to a large body of research on survey methods^35,36^. Sleep problems and ADHD screening positive were shown to be strongly correlated in our study (OR = 2.12). This is in line with other studies conducted on medical students, which found that reporting symptoms of ADHD is considerably more likely when sleep quality is poor (OR = 2.21, *p* < 0.001)^39–^^42^. These results imply that executive dysfunction may be made worse by sleep loss, which would raise scores on self-report measures like the ASRS.

### Strengths and limitations of the study

Our study has several strengths. It is the first study to assess the prevalence of ADHD Symptoms and associated factors among students at universities in Egypt. We used a validated tool, the ASRS, due to its simplicity and cost-effectiveness, to assess ADHD symptoms. We collected data on a wide range of sociodemographic characteristics, which allowed us to identify factors that were associated with ADHD. We were using a convenience sampling method, which allowed us to recruit a large sample size. In addition, it was timely and important, as it can help to raise awareness of ADHD and inform the development of effective prevention and treatment strategies. Finallywe used a clear description of regression modeling, which gave more strength for our study.

However, our study has a few limitations. Its cross-sectional nature cannot establish causality. Our survey was vulnerable to recall bias, which can lead to underestimating prevalence. Furthermore, as the ASRS is a screening rather than a diagnostic tool, the lack of formal clinical interviews hindered our ability to make a proven clinical diagnosis of ADHD.

Nevertheless, they do not necessarily invalidate the study. Cross-sectional studies can still provide valuable information about the prevalence of ADHD Symptoms and associated factors.

In addition, the sampling approach used in the study limits the generalizability of our findings. Convenience sampling as an online recruitment strategy reduces external validity and increases the risk of selection bias. Because of the notable overrepresentation of female students (65%) and health-related and medical courses (74%), our study sample is not strictly representative of all Egyptian university students.

Additionally, the cumulative academic achievement (GPA) was not included in our study to avoid recall bias and prevent missing data from participants who might not accurately remember their scores.

### Recommendations

Based on these limitations, we recommend more future well-designed studies about adult ADHD to clarify the nature in adults, its etiology, and pathophysiology. Also, we need to explain why ADHD symptoms decline with age, as it is still unclear. Nevertheless, we should think about the validation of applying children’s ADHD criteria in the diagnosis of adult ADHD^34^. We should make standardization of diagnostic tools in upcoming studies to avoid this huge variation in estimating prevalence and associated factors.

Regarding external validity, some points need to be addressed in further research. Although we have a large sample size (2689 participants), 32.8% were males, which indicates a gender imbalance. This could affect generalizability. We collected data from students at the university, so the median age was a mean age of 21 years (SD = 2), indicating that the sample is relatively young. This may affect generalizability to older people. We conducted our study in Egypt. Based on our cultural, environmental, and medical system differences, our results can be different in another region or country. The lack of significant regional differences within Egypt is noted, but this does not address differences between Egypt and other countries. We used specific cutoff points (≥ 14) to identify suspected ADHD cases. Comparing these diagnostic criteria and tools with those used in other studies or clinical settings can ensure consistency. The ADHD assessment method (e.g., self-reported questionnaires, clinical interviews) can affect the results. To get more precise estimates of the prevalence of ADHD in adults, future research should make use of established diagnostic instruments and structured clinical interviews. We recommend that future papers include objective academic metrics (GPA) in addition to standardized nutritional assessments to better clarify the suspected relationships.

## Conclusion

This study found a high prevalence of ADHD symptom screening positivity among Egyptian university students using the ASRS tool. Several psychosocial and lifestyle factors were independently associated with positive screening results, particularly sleep disorders, childhood trauma, smoking, and mental health conditions. Given the use of a screening instrument and non-representative sampling, these findings should not be interpreted as diagnostic prevalence estimates. Further studies using structured clinical interviews and representative sampling are needed.

## Data Availability

Data will be provided upon request from the corresponding author“Sara Hosny El-Farargy”dr.sara.hosny2020@gmail.com.
